# Unbiased estimation in seamless phase II/III trials with unequal treatment effect variances and hypothesis‐driven selection rules

**DOI:** 10.1002/sim.6974

**Published:** 2016-04-21

**Authors:** David S. Robertson, A. Toby Prevost, Jack Bowden

**Affiliations:** ^1^ MRC Biostatistics UnitCambridgeU.K.; ^2^ Imperial College LondonLondonU.K.; ^3^ MRC Integrative Epidemiology UnitUniversity of BristolBristolU.K.

**Keywords:** adaptive seamless designs, phase II/III clinical trials, treatment selection, uniformly minimum variance unbiased estimator

## Abstract

Seamless phase II/III clinical trials offer an efficient way to select an experimental treatment and perform confirmatory analysis within a single trial. However, combining the data from both stages in the final analysis can induce bias into the estimates of treatment effects. Methods for bias adjustment developed thus far have made restrictive assumptions about the design and selection rules followed. In order to address these shortcomings, we apply recent methodological advances to derive the uniformly minimum variance conditionally unbiased estimator for two‐stage seamless phase II/III trials. Our framework allows for the precision of the treatment arm estimates to take arbitrary values, can be utilised for all treatments that are taken forward to phase III and is applicable when the decision to select or drop treatment arms is driven by a multiplicity‐adjusted hypothesis testing procedure. © 2016 The Authors. Statistics in Medicine Published by John Wiley & Sons Ltd.

## 1 Introduction

Seamless phase II/III designs are key examples of adaptive clinical trials where data from a learning phase and a confirmatory phase are combined to promote efficient drug development. Typically, such trials will have two stages separated by an interim analysis. In stage 1, which resembles a traditional phase II trial, multiple experimental treatments or drug doses are simultaneously compared against a control. The most promising candidates are then selected for confirmatory analysis in stage 2, which corresponds to a phase III trial.

Recent examples of seamless phase II/III trials in clinical practice include dose selection for chronic obstructive pulmonary disorder 1, acute myocardial infarction 2 and treatment selection for colorectal cancer 3. Regulatory guidance dealing with such adaptive trial designs has been produced in Europe by the European Medicines Agency 4 and in the USA by the Food and Drug Administration 5.

Unlike the classical approach where only phase III patients contribute to the confirmatory analysis, in seamless phase II/III trials, the final analysis utilises data from both stages. Whilst combining the data is efficient in terms of time and resources (and of course in a purely statistical sense too), it can inflate the type I error of hypothesis tests and induce bias into the naïve estimates of treatment effect, because of the dual influence of multiplicity and selection 6.

In this paper, we consider point estimation of the treatment effects. In particular, our focus is on conditionally unbiased estimation using the method of Rao–Blackwellization. Briefly, this involves taking the unbiased stage 2 data and conditioning on a complete, sufficient statistic. The resulting estimator is the uniformly minimum variance conditionally unbiased estimator (UMVCUE).

Our key starting point in deriving the UMVCUE for the treatment effect is the paper by Kimani *et al.* 7. Building on the seminal framework of Cohen and Sackorwitz 8, Kimani *et al.* derived the UMVCUEs for the means of the selected and control treatments separately and then took the difference to give an unbiased estimator for the treatment difference. However, a number of limitations to this approach currently exist.

Firstly, the methodology does not try to explicitly take into account differing treatment effect variances. Hence, if treatment selection is based on standardised differences, the estimator will not necessarily be unbiased when the treatment effect variances are unequal. Many other authors have also used the convention of equal variances in order to derive their results 9, 10, 11, 12. This allows the information from the treatment and control arms at phases II and III to be separated, and all estimates can be assumed to be independent. However, there are many reasons why differences in the treatment effect variances may occur, even if this was not planned at the outset. For example, there may be unequal drop out across arms, or there simply may be true differences between the variance of patient outcomes for different experimental treatments.

Secondly, the estimator is only for the treatment with the largest treatment difference. However, we may be interested in estimating the treatment difference for several treatments, for example, when the decision to select/drop treatments is driven by a formal hypothesis testing procedure. We note that formulae that are applicable to this setting have been derived by Bowden and Glimm 10, but for different ranking and selection rules to those considered in this paper. In practice, and as for any confirmatory trial, hypothesis testing (with rigid type I error control) will be the primary focus of a seamless phase II/III trial, with estimation being an important but secondary target. For a comprehensive overview of the methodology for hypothesis testing in seamless phase II/III trials, we refer the reader to the reviews of Bretz *et al.* 13 and Stallard and Todd 14.

In this paper, we aim to address these limitations, by transferring recent methodological advancements in UMVCUEs for multivariate normal outcomes proposed by Robertson *et al.* 15 to the seamless phase II/III setting. We derive formulae that are applicable in full generality for the *j*th‐ranked treatment where the precision of treatment arm estimates can take arbitrary values.

The rest of the paper is organised as follows. In Section 2, we describe the set‐up and notation, derive the UMVCUE for the maximum treatment difference and compare it analytically with the Kimani *et al.* estimator. We carry out a simulation study in Section 3 to compare the bias and mean square error of the Kimani *et al.* estimator and our UMVCUE in a variety of trial settings. Section 4 describes how our UMVCUE can be used in the context of a seamless phase II/III trial where a multiplicity adjusted hypothesis procedure drives the design and is illustrated with a simple practical example. We discuss all of our results in Section 5 and consider future avenues of research.

## 2 Framework for the uniformly minimum variance conditionally unbiased estimator

We use the adaptive seamless design (ASD) setting of Kimani *et al.* 7 as our starting point. Consider an ASD with two stages, where stage 1 is used to select the most promising treatment and stage 2 is used for confirmatory analysis. Let *K*( ≥2) denote the number of experimental treatments tested in stage 1 for comparison with the control. The treatment that shows the highest standardised treatment difference (as defined in the succeeding text) in stage 1 is then selected to continue to stage 2, along with the control.

We now allow for the treatment arm estimates to have unequal variances. Let *n*_1*i*_ denote the number of subjects allocated to treatment *i* (*i* = 0,1,…,*K*) in stage 1, where *i* = 0 corresponds to the control treatment. We assume that the stage 1 sample mean for treatment *i*, denoted *X*_*i*_, is normally distributed with unknown mean *μ*_*i*_ and known variance $\sigma_{1i}^{2}$. As an example, if we also assume that there is a known common variance *σ* ^2^ across the treatment groups, then $\sigma_{1i}^{2}=\sigma^{2}/n_{1i}$.

At the end of stage 1, we rank the treatments according to their standardised treatment difference. More explicitly, we rank treatment *i* above treatment *j* if

(1) $$\frac{X_{i}-X_{0}}{\sqrt{\mathrm{Var}(X_{i}-X_{0})}}>\frac{X_{j}-X_{0}}{\sqrt{\mathrm{Var}(X_{j}-X_{0})}}\;\Rightarrow \;\frac{X_{i}-X_{0}}{\sqrt{\sigma_{1i}^{2}+\sigma_{10}^{2}}}>\frac{X_{j}-X_{0}}{\sqrt{\sigma_{1j}^{2}+\sigma_{10}^{2}}}.$$

In contrast, in the Kimani *et al.* setting, we rank the treatments by the stage 1 sample means, and so treatment *i* is ranked above treatment *j* if *X*_*i*_>*X*_*j*_. Note that if we have a common stage 1 variance, that is, *σ*_1*i*_=*σ*_1_ for *i* = 1,…,*K*, then the two ranking rules are the same, because when ranking by standardised treatment difference, the denominator and control data *X*_0_ can be ignored.

We let the treatment with the highest ranking be denoted *S* (*S*∈{1,…,*K*}), where *S* is a random variable. We also allow early stopping of the trial for futility: the trial continues to stage 2 if $\frac{X_{S}-X_{0}}{\sqrt{\sigma_{1S}^{2}+\sigma_{10}^{2}}}>b$, where *b* is a (pre‐specified and constant) futility boundary.

For notational convenience, let Θ_*i*_=*X*_*i*_−*X*_0_ denote the stage 1 sample mean treatment difference for treatment *i* (*i* = 1,…,*K*) and define $\lambda_{i}=1/\sqrt{\sigma_{1i}^{2}+\sigma_{10}^{2}}$ (*i* = 1,…,*K*). Then treatment *i* is ranked above treatment *j* if *λ*_*i*_Θ_*i*_>*λ*_*j*_Θ_*j*_. As well, the futility boundary implies that *λ*_*S*_Θ_*S*_>*b* in order for the trial to continue to stage 2.

If the trial continues to stage 2, then let *n*_2*i*_ denote the number of subjects allocated to treatment *i* (*i* = 0,*S*). We assume that the stage 2 sample means, denoted *Y*_*i*_, follow a $N(\mu_{i},\sigma_{2i}^{2})$ distribution. As before, if we also assume a known common variance *σ* ^2^, then $\sigma_{2i}^{2}=\sigma^{2}/n_{2i}$. We can define the selection time for treatment *i* (*i* = 0,*S*) as $t_{i}=\sigma_{2i}^{2}/(\sigma_{1i}^{2}+\sigma_{2i}^{2})$. Hence, the sample mean from the two stages for the control is *Z*_0,MLE_=*t*_0_ *X*_0_+(1 − *t*_0_)*Y*_0_ and similarly *Z*_*S*,MLE_=*t*_*S*_ *X*_*S*_+(1 − *t*_*S*_)*Y*_*S*_ for the selected treatment.

After the trial is completed, the aim is to estimate the treatment difference *θ*_*S*_=*μ*_*S*_−*μ*_0_. As Kimani *et al.* note, the maximum likelihood estimator (MLE) for *θ*_*S*_ is *D*_*S*,MLE_=*Z*_*S*,MLE_−*Z*_0,MLE_. This estimator will likely be biased, because it does not take into account the selection rules. An unbiased estimator can easily be found by just using the stage 2 data, because *Y*_*S*_ and *Y*_0_ are unbiased estimators for *μ*_*S*_ and *μ*_0_, respectively. Hence, the sample difference *Y* = *Y*_*S*_−*Y*_0_ is an unbiased estimator for *θ*_*S*_. However, this estimator will be inefficient because it does not use the stage 1 data.

### 2.1 Calculating the uniformly minimum variance conditionally unbiased estimator

Using the theory from the general multivariate normal setting 15, we derive the UMVCUE for this framework. The stage 1 sample mean treatment differences Θ_*i*_=*X*_*i*_−*X*_0_ are normally distributed: $\Theta_{i}∼N\left(\mu_{i}-\mu_{0},\sigma_{1i}^{2}+\sigma_{10}^{2}\right)$. Because **Θ** = (Θ_1_,…,Θ_*K*_) is a linear transformation of ***X*** = (*X*_0_,*X*_1_,…,*X*_*K*_), then **Θ** follows a multivariate normal distribution with mean ***θ*** = (*θ*_1_,…,*θ*_*K*_) and covariance matrix Σ, where *θ*_*i*_=*μ*_*i*_−*μ*_0_ and Σ_*ij*_=Cov(Θ_*i*_,Θ_*j*_). Hence,

$$\begin{array}{cc} \Sigma_{\mathrm{ii}} & =\sigma_{1i}^{2}+\sigma_{10}^{2}\;i\in \{1,\ldots ,K\}\\ \Sigma_{\mathrm{ij}} & =\sigma_{10}^{2}\;i,j\in \{1,\ldots ,K\}\;,\;i\neq j. \end{array}$$

The stage 2 sample mean treatment difference *Y* = *Y*_*S*_−*Y*_0_ is also normally distributed with $Y\;∼\;N(\mu_{S}\;-\mu_{0},\sigma_{20}^{2}+\sigma_{2S}^{2})$. Let *Q* be the event {**Θ**:*λ*_1_Θ_1_>*λ*_2_Θ_2_>⋯>*λ*_*K*_Θ_*K*_,*λ*_1_Θ_1_>*b*}, which implies that the trial continues to stage 2 and that treatment *i* has rank *i*, with *S* = 1. Without loss of generality, we condition on *Q* for the remainder of this section. For notational convenience, let $\nu^{2}=\sigma_{10}^{2}+\sigma_{11}^{2}$ and $\tau^{2}=\sigma_{20}^{2}+\sigma_{21}^{2}$. Then the statistics ***Z*** = (*Z*_1_,…,*Z*_*K*_) are sufficient and complete for ***θ***, where

(2) $$\begin{array}{cc} Z_{1} & =\Theta_{1}+\frac{\nu^{2}}{\tau^{2}}Y\\ Z_{i} & =\Theta_{i}+\frac{\sigma_{10}^{2}}{\tau^{2}}Y\;i=2,\ldots ,K. \end{array}$$

Using the notation defined previously, we have the following form for the UMVCUE, with a proof provided in Appendix A.1.

Theorem 2.1 The UMVCUE for *θ*_1_=*μ*_1_−*μ*_0_ given *Q* is

(3) $$Û=\frac{\tau^{2}Z_{1}}{\nu^{2}+\tau^{2}}-\frac{\tau^{2}}{\sqrt{\nu^{2}+\tau^{2}}}\frac{\phi (W_{1})-\phi (W_{2})}{\Phi (W_{1})-\Phi (W_{2})}\;\;,$$

where

$$\begin{array}{cc} W_{i} & =\frac{k_{i}\sqrt{\nu^{2}+\tau^{2}}}{\tau^{2}}-\frac{Z_{1}}{\sqrt{\nu^{2}+\tau^{2}}}\;\text{for}i=1,2\;\;;\\ k_{1} & =\min(A_{1},A_{2},A_{3}),\;k_{2}=\max(A_{4},A_{5}),\\ A_{1} & =\frac{\tau^{2}}{\nu^{2}}\left(Z_{1}-\frac{b}{\lambda_{1}}\right),\\ A_{2} & =\left\{\frac{\tau^{2}\left(\lambda_{1}Z_{1}-\lambda_{2}Z_{2}\right)}{\sigma_{10}^{2}\left(\lambda_{1}-\lambda_{2}\right)+\lambda_{1}\sigma_{11}^{2}}:\lambda_{1}\sigma_{11}^{2}>\left(\lambda_{2}-\lambda_{1}\right)\sigma_{10}^{2}\right\},\\ A_{3} & =\left\{\frac{\tau^{2}\left(\lambda_{i}Z_{i}-\lambda_{i+1}Z_{i+1}\right)}{\sigma_{10}^{2}\left(\lambda_{i}-\lambda_{i+1}\right)}:\sigma_{1,i+1}^{2}>\sigma_{1i}^{2}\;;\;i=2,\ldots ,K-1\right\},\\ A_{4} & =\left\{\frac{\tau^{2}\left(\lambda_{1}Z_{1}-\lambda_{2}Z_{2}\right)}{\sigma_{10}^{2}\left(\lambda_{1}-\lambda_{2}\right)+\lambda_{1}\sigma_{11}^{2}}:\lambda_{1}\sigma_{11}^{2}<\left(\lambda_{2}-\lambda_{1}\right)\sigma_{10}^{2}\right\},\\ A_{5} & =\left\{\frac{\tau^{2}\left(\lambda_{i}Z_{i}-\lambda_{i+1}Z_{i+1}\right)}{\sigma_{10}^{2}\left(\lambda_{i}-\lambda_{i+1}\right)}:\sigma_{1,i+1}^{2}<\sigma_{1i}^{2}\;;\;i=2,\ldots ,K-1\right\}, \end{array}$$

and we define min({*∅*}) =+ *∞* and max({*∅*}) =− *∞*.

Note that the first term in expression (3), namely, $\frac{\tau^{2}Z_{1}}{\nu^{2}+\tau^{2}}$, is equal to the MLE *D*_*S*,MLE_.

### 2.2 Comparison with the estimator of Kimani et al.

Suppose we set $\sigma_{1i}^{2}=\sigma_{1}^{2}$(for *i* = 0,1,…,*K*) and $\sigma_{2i}^{2}=\sigma_{2}^{2}$(for *i* = 0,1). We then recover the setting of Kimani *et al.* 7, so we can compare our results. In this case, ranking by standardised treatment difference reduces down to ranking by the stage 1 sample mean, in the sense that they always select the same treatment. Kimani *et al.* derived the following unbiased estimator for *θ*_1_=*μ*_1_−*μ*_0_:

(4) $$\begin{array}{cc} D_{1,\text{CHN}} & =Z_{1,\text{CHN}}-Z_{0,\text{CHN}}\\ =\left[\frac{\sigma_{2}^{2}X_{1}+\sigma_{1}^{2}Y_{1}}{\sigma_{1}^{2}+\sigma_{2}^{2}}-\frac{\sigma_{2}^{2}}{\sqrt{\sigma_{1}^{2}+\sigma_{2}^{2}}}\frac{\phi (W_{B})}{\Phi (W_{B})}\right]-\left[\frac{\sigma_{2}^{2}X_{0}+\sigma_{1}^{2}Y_{0}}{\sigma_{1}^{2}+\sigma_{2}^{2}}+\frac{\sigma_{2}^{2}}{\sqrt{\sigma_{1}^{2}+\sigma_{2}^{2}}}\frac{\phi \left(W_{B_{1}}\right)}{\Phi \left(W_{B_{1}}\right)}\right]\\ =\frac{\sigma_{2}^{2}(X_{1}-X_{0})+\sigma_{1}^{2}(Y_{1}-Y_{0})}{\sigma_{1}^{2}+\sigma_{2}^{2}}-\frac{\sigma_{2}^{2}}{\sqrt{\sigma_{1}^{2}+\sigma_{2}^{2}}}\left[\frac{\phi (W_{B})}{\Phi (W_{B})}+\frac{\phi \left(W_{B_{1}}\right)}{\Phi \left(W_{B_{1}}\right)}\right]\;\;, \end{array}$$

where

$$\begin{array}{cc} W_{B} & =\frac{\sqrt{\sigma_{1}^{2}+\sigma_{2}^{2}}}{\sigma_{1}^{2}}\left(\frac{\sigma_{2}^{2}X_{1}+\sigma_{1}^{2}Y_{1}}{\sigma_{1}^{2}+\sigma_{2}^{2}}-\max\{X_{0}+b\sigma_{1}\sqrt{2},X_{2}\}\right)\\ W_{B_{1}} & =\frac{\sqrt{\sigma_{1}^{2}+\sigma_{2}^{2}}}{\sigma_{1}^{2}}\left(X_{1}-b\sigma_{1}\sqrt{2}-\frac{\sigma_{2}^{2}X_{0}+\sigma_{1}^{2}Y_{0}}{\sigma_{1}^{2}+\sigma_{2}^{2}}\right). \end{array}$$

As for the UMVCUE derived in Section 2, firstly note that *σ*_1,*j* + 1_=*σ*_1*j*_=*σ*_1_ for *j* = 2,…,*K* − 1. Hence, the sets *A*_3_ and *A*_5_ are empty in Equation (3). In addition, $\sigma_{11}^{2}=\sigma_{12}^{2}\;\Rightarrow \;\lambda_{1}=\lambda_{2}$, and hence, *A*_4_ is also empty. Note also that $\tau^{2}=2\sigma_{2}^{2}$ and $\nu^{2}=2\sigma_{1}^{2}$.

The sufficient statistics in this case are

$$\begin{array}{cc} Z_{1} & =\Theta_{1}+\frac{\sigma_{1}^{2}}{\sigma_{2}^{2}}Y\\ Z_{i} & =\Theta_{i}+\frac{\sigma_{1}^{2}}{2\sigma_{2}^{2}}Y\;i=2,\ldots ,K. \end{array}$$

Hence, the UMVCUE equals

(5) $$\begin{array}{cc} Û & =\frac{\sigma_{2}^{2}Z_{1}}{\sigma_{1}^{2}+\sigma_{2}^{2}}-\frac{\sigma_{2}^{2}\sqrt{2}}{\sqrt{\sigma_{1}^{2}+\sigma_{2}^{2}}}\frac{\phi (W)}{\Phi (W)}\\ =\frac{\sigma_{2}^{2}(X_{1}-X_{0})+\sigma_{1}^{2}(Y_{1}-Y_{0})}{\sigma_{1}^{2}+\sigma_{2}^{2}}-\frac{\sigma_{2}^{2}\sqrt{2}}{\sqrt{\sigma_{1}^{2}+\sigma_{2}^{2}}}\frac{\phi (W)}{\Phi (W)}\;\;, \end{array}$$

where

$$\begin{array}{cc} W & =\frac{k\sqrt{\sigma_{1}^{2}+\sigma_{2}^{2}}}{\sigma_{2}^{2}\sqrt{2}}-\frac{Z_{1}}{\sqrt{2\left(\sigma_{1}^{2}+\sigma_{2}^{2}\right)}},\\ k\;\; & =\min(A_{1},A_{2}),\\ A_{1} & =\frac{\sigma_{2}^{2}}{\sigma_{1}^{2}}\left(Z_{1}-b\sigma_{1}\sqrt{2}\right),\;A_{2}=\frac{2\sigma_{2}^{2}}{\sigma_{1}^{2}}(Z_{1}-Z_{2}). \end{array}$$

Now, we can rewrite *W* as

$$\begin{array}{cc} W & =\frac{\sqrt{\sigma_{1}^{2}+\sigma_{2}^{2}}}{\sigma_{2}^{2}\sqrt{2}}\min\left(\frac{2\overset{2}{\underset{2}{\sigma }}}{\overset{2}{\underset{1}{\sigma }}}\left(Z_{1}-Z_{2}\right),\frac{\overset{2}{\underset{2}{\sigma }}}{\overset{2}{\underset{1}{\sigma }}}\left(Z_{1}-b\sigma_{1}\sqrt{2}\right)\right)-\frac{Z_{1}}{\sqrt{2\left(\overset{2}{\underset{1}{\sigma }}+\overset{2}{\underset{2}{\sigma }}\right)}}\\ =\frac{\sqrt{\sigma_{1}^{2}+\sigma_{2}^{2}}}{\sigma_{1}^{2}\sqrt{2}}\left[\frac{\sigma_{2}^{2}Z_{1}}{\sigma_{1}^{2}+\sigma_{2}^{2}}-\max\left(2Z_{2}-Z_{1},b\sigma_{1}\sqrt{2}\right)\right]\\ =\frac{\sqrt{\sigma_{1}^{2}+\sigma_{2}^{2}}}{\sigma_{1}^{2}\sqrt{2}}\left[\frac{\sigma_{2}^{2}\left(X_{1}-X_{0}\right)+\sigma_{1}^{2}\left(Y_{1}-Y_{0}\right)}{\sigma_{1}^{2}+\sigma_{2}^{2}}-\max\left\{2\left(X_{2}-X_{1}\right)-\left(X_{1}-X_{0}\right),b\sigma_{1}\sqrt{2}\right\}\right]. \end{array}$$

Even for the special case when the two methods always select the same treatment, the estimators are not equal, because the estimators condition on different selection rules and data. We return to this issue in Section 3.1.1.

## 3 Simulation study

We now perform a simulation study to explore the bias and mean squared error (MSE) of the estimators described in Section 2. Because the performance of the Kimani *et al.* (*D*_1,CHN_), naïve (*D*_1,MLE_) and stage 2 (*D*_1,2_) estimators have already been extensively studied in 7, we focus on comparing the properties of our UMVCUE with these existing estimators.

### 3.1 Equal variances

Initially we use the setting of Kimani *et al.* 7, with a common variance *σ* ^2^, *n*_1*i*_=*n*_1_(*i* = 0,1,…,*K*) and *n*_2*i*_=*n*_2_(*i* = 0,1). Hence, the stages 1 and 2 variances are all equal, and we can write $\sigma_{1i}^{2}=\sigma_{1}^{2}=\sigma^{2}/n_{1}$ and $\sigma_{2i}^{2}=\sigma_{2}^{2}=\sigma^{2}/n_{2}$. Also the selection times *t*_*i*_ all equal $t=\frac{n_{1}}{n_{1}+n_{2}}$.

In our simulations, we set the common variance *σ* = 1 and vary the selection time point *t* in the interval (0,1). Because the stages 1 and 2 sample sizes per arm are equal, we can present the bias and the $\sqrt{\text{MSE}}$ of the estimators in units of the standard error (SE) $\sqrt{2/(n_{1}+n_{2})}$. This is the standard deviation for the difference of a single experimental treatment–control comparison and makes the results invariant to sample size 7.

**Figure 1:**
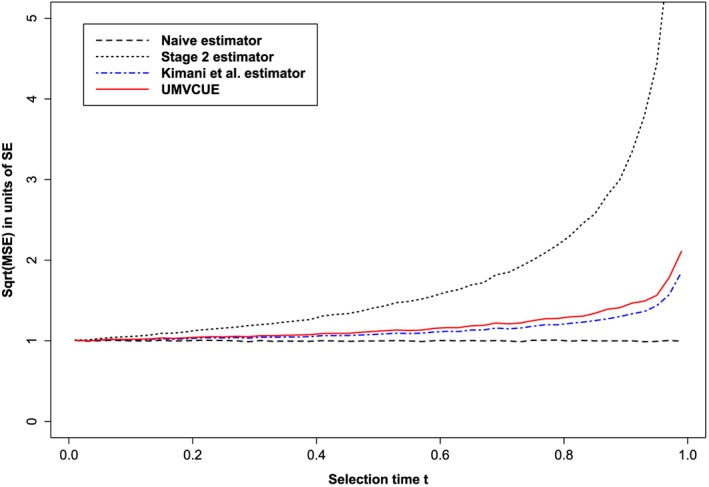
$\sqrt{\text{MSE}}$ for various estimators, in units of standard error (SE). We set *μ*_0_=0,*μ*_1_=*μ*_2_=0.05 and *b* =− *∞*. There were 20000 simulated trials for each value of the selection time *t*. MSE, mean squared error; UMVCUE, uniformly minimum variance conditionally unbiased estimator.

Figure 1 shows the $\sqrt{\text{MSE}}$ when the number of experimental treatments *K* = 2 and *μ*_0_=0,*μ*_1_=*μ*_2_=0.05. We assume there is no early stopping for futility, which corresponds to the futility boundary *b* =− *∞*. Note that we do not give a plot of the bias because (as expected) the bias of our UMVCUE (as well as the Kimani estimator) is not noticeably different from zero in the simulations. The MSE of the Kimani estimator and UMVCUE are approximately equal, but for all values of *t*, the UMVCUE has a higher MSE. This difference is an increasing function of *t*.

Table 1 shows the bias and $\sqrt{\text{MSE}}$(in units of SE) for a range of representative parameter values, with the selection time *t* = 0.5. As expected, the UMVCUE is unbiased in its mean in all cases. The UMVCUE still has a slightly higher $\sqrt{\text{MSE}}$ – although it is within 10% of the $\sqrt{\text{MSE}}$ for the Kimani estimator. This difference is a decreasing function of the futility boundary *b* (or equivalently, an increasing function of the probability of early stopping for futility).

**Table 1:** Simulation results for *t* = 0.5. There were 100000 simulations for each set of parameter values.

	Bias $\left(\sqrt{\text{MSE}}\right)$ in units of SE			
Parameter values	Naïve	Stage 2	Kimani	UMVCUE
*μ*_0_=0,*μ*_1_=*μ*_2_=0.05	0.286	0.002	0.003	0.003
*b* =− *∞*	(1.002)	(1.412)	(1.085)	(1.119)
*μ*_0_=0,*μ*_1_=*μ*_2_=0.05	0.511	−0.008	−0.003	−0.004
*b* = 0	(1.002)	(1.407)	(1.188)	(1.198)
*μ*_0_=0.1,*μ*_1_=*μ*_2_=0.3	0.276	−0.005	−0.007	−0.006
*b* =− *∞*	(0.997)	(1.419)	(1.083)	(1.119)
*μ*_0_=0.1,*μ*_1_=*μ*_2_=0.3	0.330	0.004	0.004	0.005
*b* = 0.1	(0.986)	(1.413)	(1.111)	(1.140)
*μ*_0_=0.05,*μ*_1_=0.15,*μ*_2_=0.1	0.439	−0.004	−0.003	−0.004
*b* = 0	(0.985)	(1.414)	(1.166)	(1.181)
*μ*_0_=0,*μ*_1_=*μ*_2_=*μ*_3_=*μ*_4_=0.05	0.650	0.005	0.005	0.003
*b* = 0.05	(1.087)	(1.412)	(1.186)	(1.222)

MSE, mean squared error; UMVCUE, uniformly minimum variance conditionally unbiased estimator.

#### 3.1.1 How can the UMVCUE be worse in terms of MSE?

The result that the UMVCUE has a higher MSE than the Kimani estimator seems somewhat counter‐intuitive and indeed seemingly in contradiction of the very definition of the UMVCUE we have derived. However, the explanation is that the two estimators are using different amounts of data. The Kimani estimator is a function of the individual treatment mean outcome statistics *X*_0_,*X*_1_,…,*X*_*K*_,*Y*_0_,*Y*_1_, whereas our UMVCUE is a function of *X*_1_−*X*_0_,…,*X*_*K*_−*X*_0_,*Y*_1_−*Y*_0_. That is, we are not explicitly using the control data *X*_0_,*Y*_0_ in the UMVCUE – all we need are the treatment *differences* in both stages. In the special case of equal variances, for which the experimental and control group data *can* be separated, this loss of information results in a slightly greater MSE for the UMVCUE. We return to this issue in the discussion.

### 3.2 Unequal variances

We have seen that the Kimani estimator performs well when the stages 1 and 2 variances are equal. We now explore what happens when this assumption no longer holds – that is, when the *σ*_1*i*_ and *σ*_2*i*_ are distinct. In this setting, ranking by standardised treatment difference no longer reduces down to ranking by the stage 1 sample mean. This means that the selection based on standardised observed differences will not necessarily select the treatment with the highest stage 1 sample mean. The Kimani estimator will overcorrect for bias in this setting, because it assumes that we are *always* selecting the treatment with the highest treatment effect.

We now conduct simulation studies to see to what extent the Kimani estimator is appropriate for the selection rule that uses standardised treatment differences. Although the Kimani estimator is being incorrectly applied in this setting, because it slightly outperformed the UMVCUE in terms of MSE when the variances are equal, it is interesting to investigate whether it does so again.

We can straightforwardly modify the Kimani estimator to take into account the differing variances in stages 1 and 2 as follows:

$$\begin{array}{cc} D_{1,\text{CHN}} & =Z_{1,\text{CHN}}-Z_{0,\text{CHN}}\\ =\left[\frac{\sigma_{21}^{2}X_{1}+\sigma_{11}^{2}Y_{1}}{\sigma_{11}^{2}+\sigma_{21}^{2}}-\frac{\sigma_{21}^{2}}{\sqrt{\sigma_{11}^{2}+\sigma_{21}^{2}}}\frac{\phi (W_{B})}{\Phi (W_{B})}\right]-\left[\frac{\sigma_{20}^{2}X_{0}+\sigma_{10}^{2}Y_{0}}{\sigma_{10}^{2}+\sigma_{20}^{2}}+\frac{\sigma_{20}^{2}}{\sqrt{\sigma_{10}^{2}+\sigma_{20}^{2}}}\frac{\phi \left(W_{B_{1}}\right)}{\Phi \left(W_{B_{1}}\right)}\right]\;\;, \end{array}$$

where

$$\begin{array}{cc} W_{B} & =\frac{\sqrt{\sigma_{11}^{2}+\sigma_{21}^{2}}}{\sigma_{11}^{2}}\left(\frac{\sigma_{21}^{2}X_{1}+\sigma_{11}^{2}Y_{1}}{\sigma_{11}^{2}+\sigma_{21}^{2}}-\max\left\{X_{0}+b\sqrt{\sigma_{11}^{2}+\sigma_{10}^{2}},X_{2}\right\}\right)\\ W_{B_{1}} & =\frac{\sqrt{\sigma_{10}^{2}+\sigma_{20}^{2}}}{\sigma_{10}^{2}}\left(X_{1}-b\sqrt{\sigma_{11}^{2}+\sigma_{10}^{2}}-\frac{\sigma_{20}^{2}X_{0}+\sigma_{10}^{2}Y_{0}}{\sigma_{10}^{2}+\sigma_{20}^{2}}\right). \end{array}$$

**Figure 2:**
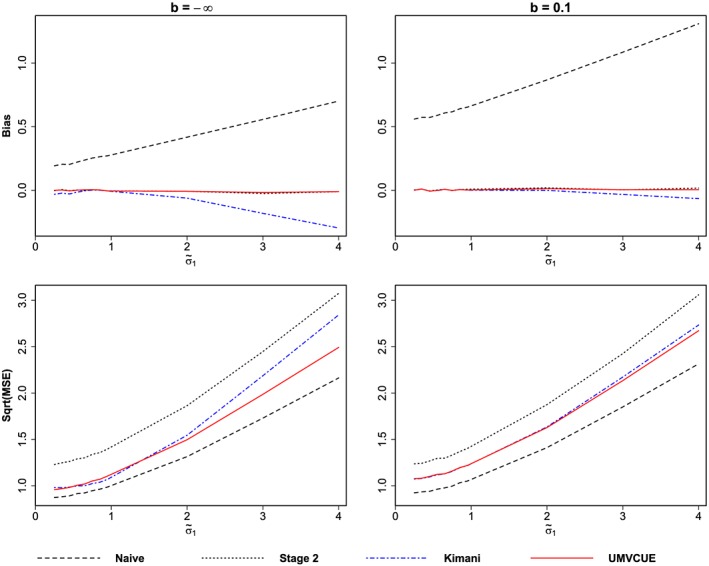
Bias and $\sqrt{\text{MSE}}$ for the estimators, using individual variances for the Kimani estimator. We set *μ*_0_=0, *μ*_1_=*μ*_2_=0.05, *σ*_21_=*σ*_11_ and *σ*_10_=*σ*_20_=1. There were 50000 simulated trials for each value of ${\tilde{\sigma }}_{1}$.

Consider now the scenario where *K* = 2 and one of the experimental treatments has variance ${\tilde{\sigma }}_{1}^{2}$ say, whereas the other experimental treatment and the control both have variance equal to 1. Figure 2 shows the (unadjusted) bias and $\sqrt{\text{MSE}}$ for the various estimators where we vary ${\tilde{\sigma }}_{1}$ from 0.25 to 4. We also set *σ*_21_=*σ*_11_, whilst keeping *σ*_20_=1. Note that $\sigma_{11}^{2}$ is the variance of the treatment that is selected to continue to stage 2.

If we assume a common variance *σ* ^2^ across treatment groups, then values of ${\tilde{\sigma }}_{1}>2$(or <0.5) imply unrealistic unequal allocations to the treatment groups that would rarely occur in practice. However, such scenarios could occur where there is reason to believe treatment 1 has a different treatment effect variance from the other treatment (and the control) after looking at previous trial or pilot study data. This may make biological sense too, if treatment 1 is a different class of drug to the others. In that case, we could have ${\tilde{\sigma }}_{1}\;>\;2$ despite having equal allocation ratios to the treatment groups and the control.

As expected, the stage 2 estimator and UMVCUE are unbiased for all values of ${\tilde{\sigma }}_{1}$, whilst the naïve estimator is positively biased. However, we see that for ${\tilde{\sigma }}_{1}\neq 1$, the Kimani estimator is negatively biased, with the bias much worse when *b* =− *∞* compared with *b* = 0.1. This negative bias steadily increases as ${\tilde{\sigma }}_{1}$ increases above 1. Indeed, when ${\tilde{\sigma }}_{1}=4$, the Kimani estimator has a substantial bias when *b* =− *∞*(which corresponds to an early stopping probability of 0).

In terms of the MSE, as expected the naïve estimator has the lowest MSE. The Kimani estimator has a higher MSE than the UMVCUE except when ${\tilde{\sigma }}_{1}$ is close to 1. There is a steady increase in the Kimani estimator's MSE for ${\tilde{\sigma }}_{1}>1$. The MSE of the UMVCUE is slightly higher when *b* = 0.1 compared with *b* =− *∞* and vice‐versa for the MSE of the Kimani estimator.

## 4 Unbiased estimation for hypothesis‐driven designs

Finally, we look at the application of our estimator within the context of formal hypothesis testing. We illustrate this with an example based on the case study in 16. Suppose we are comparing three experimental drugs with a placebo for the treatment of generalised anxiety disorder. We assume that the outcomes (the total score on the Hamilton Rating Scale for Anxiety) are normally distributed with common standard deviation *σ* = 6.

The trial is planned with equal allocations to each treatment, with *n*_1_=*n*_2_=71 subjects per group. However, suppose that the randomisation procedure used leads to an unequal number of subjects in each treatment group. Table 2 shows the observed data for both stages of the trial.

**Table 2:** Example data from a seamless phase II/III trial.

	Stage 1					Stage 2	
	*n*_1*i*_	Observed	*z*‐statistic	*p*_1*i*_		*n*_2*i*_	Observed
Placebo	70	0.4	—	—		68	−0.3
Treatment 1	72	2.2	1.787	0.0369		75	1.7
Treatment 2	68	2.4	1.958	0.0251		70	2.2
Treatment 3	74	3.2	2.799	0.0026		71	1.9

The aim is to take forward as many treatments as possible that pass a first‐stage *p*‐value futility threshold, set at *α*_0_=0.1. As we are in a multiple testing situation, we use multiplicity corrected *p*‐values and the closure principle in our analysis (see Section 4.2). Although the primary focus will be hypothesis testing, estimation of the treatment effects is an important secondary goal, and we would like unbiased estimates of the selected treatments' benefit over control at the end of the trial. This means that we need a way of estimating the treatment difference when (i) multiple treatments are taken forward to stage 2, and (ii) the treatments are not ranked using a rule that is concordant with ranking by the stage 1 sample mean alone. In these cases, the Kimani estimator cannot be used, and hence we need to extend our methodology (see succeeding text).

### 4.1 Uniformly minimum variance conditionally unbiased estimator for the *j*th‐ranked treatment

Suppose that we take forward the top *K* treatments from a larger group of *K*′. We want to find the UMVCUE for the *j*th best treatment out of *K*. Let *T*_*j*_=*Y*_*j*_−*Y*_0_ denote the stage 2 sample mean treatment differences for *j*∈{1,…,*K*}. We consider the more general early stopping rules where the *j*th‐ranked treatment proceeds to stage 2 if *λ*_*j*_(*X*_*j*_−*X*_0_) > *b*_*j*_.

For notational convenience, let $\nu_{j}^{2}=\sigma_{10}^{2}+\sigma_{1j}^{2}$ and $\tau_{j}^{2}=\sigma_{20}^{2}+\sigma_{2j}^{2}$. Then from the multivariate normal theory 15, for a given value of *j*∈{1,…,*K*} the statistic ***Z*_*j*_** = (*Z*_1*j*_,…,*Z*_*Kj*_) is sufficient and complete for ***θ***, where

$$\begin{array}{cc} Z_{\mathrm{jj}} & =\Theta_{j}+\frac{\nu_{j}^{2}}{\tau_{j}^{2}}T_{j}\\ Z_{\mathrm{ij}} & =\Theta_{i}+\frac{\sigma_{10}^{2}}{\tau_{j}^{2}}T_{j}\;i\neq j,\;i\in \{1,\ldots ,K\}. \end{array}$$

This time, we are conditioning on the modified event *Q*′, where *Q*′={**Θ**:*λ*_1_Θ_1_>⋯>*λ*_*K*_Θ_*K*_,*λ*_1_Θ_1_>*b*_1_,…,*λ*_*K*_Θ_*K*_>*b*_*K*_}. Then the UMVCUE for the *j*th‐ranked treatment (denoted ${Û}_{j}$) is shown in Theorem 4.1 with a proof provided in Appendix A.2.

Theorem 4.1 For a given value of *j*∈{1,…,*K*}, the UMVCUE for *θ*_*j*_=*μ*_*j*_−*μ*_0_ given *Q*′ is

(6) $${Û}_{j}=\frac{\tau_{j}^{2}Z_{\mathrm{jj}}}{\nu_{j}^{2}+\tau_{j}^{2}}-\frac{\tau_{j}^{2}}{\sqrt{\nu_{j}^{2}+\tau_{j}^{2}}}\frac{\phi (W_{1})-\phi (W_{2})}{\Phi (W_{1})-\Phi (W_{2})}\;\;,$$

where

$$\begin{array}{cc} W_{i} & =\frac{k_{i}\sqrt{\nu_{j}^{2}+\tau_{j}^{2}}}{\tau_{j}^{2}}-\frac{Z_{\mathrm{jj}}}{\sqrt{\nu_{j}^{2}+\tau_{j}^{2}}}\;\text{for}i=1,2\;\;;\\ k_{1} & =\min(A_{1},A_{2},A_{3},A_{4},A_{5}),\;k_{2}=\max(A_{6},A_{7},A_{8}),\\ A_{1} & =\frac{\tau_{j}^{2}}{\nu_{j}^{2}}\left(Z_{\mathrm{jj}}-\frac{b_{j}}{\lambda_{j}}\right);\;A_{2}=\left\{\frac{\tau_{j}^{2}}{\sigma_{10}^{2}}\left(Z_{\mathrm{ij}}-\frac{b_{i}}{\lambda_{i}}\right):i\neq j,\;i\in \{1,\ldots ,K\}\right\},\\ A_{3} & =\left\{\frac{\tau_{j}^{2}\left(\lambda_{j}Z_{\mathrm{jj}}-\lambda_{j+1}Z_{j+1,j}\right)}{\sigma_{10}^{2}\left(\lambda_{j}-\lambda_{j+1}\right)+\lambda_{j}\sigma_{1j}^{2}}:\lambda_{j}\sigma_{1j}^{2}>\left(\lambda_{j+1}-\lambda_{j}\right)\sigma_{10}^{2}\;;\;j\neq K\right\},\\ A_{4} & =\left\{\frac{\tau_{j}^{2}\left(\lambda_{j}Z_{\mathrm{jj}}-\lambda_{j-1}Z_{j-1,j}\right)}{\sigma_{10}^{2}\left(\lambda_{j}-\lambda_{j-1}\right)+\lambda_{j}\sigma_{1j}^{2}}:\lambda_{j}\sigma_{1j}^{2}<\left(\lambda_{j-1}-\lambda_{j}\right)\sigma_{10}^{2}\;;\;j\neq 1\right\},\\ A_{5} & =\left\{\frac{\tau^{2}\left(\lambda_{i}Z_{\mathrm{ij}}-\lambda_{i+1}Z_{i+1,j}\right)}{\sigma_{10}^{2}\left(\lambda_{i}-\lambda_{i+1}\right)}:\sigma_{1,i+1}^{2}>\sigma_{1i}^{2}\;;\;i\in \{1,\ldots ,K-1\}/\{j-1,j\}\right\},\\ A_{6} & =\left\{\frac{\tau_{j}^{2}\left(\lambda_{j}Z_{\mathrm{jj}}-\lambda_{j+1}Z_{j+1,j}\right)}{\sigma_{10}^{2}(\lambda_{j}-\lambda_{j+1})+\lambda_{j}\sigma_{1j}^{2}}:\lambda_{j}\sigma_{1j}^{2}<\left(\lambda_{j+1}-\lambda_{j}\right)\sigma_{10}^{2}\;;\;j\neq K\right\}, \end{array}$$

$$\begin{array}{cc} A_{7} & =\left\{\frac{\tau_{j}^{2}\left(\lambda_{j}Z_{\mathrm{jj}}-\lambda_{j-1}Z_{j-1,j}\right)}{\sigma_{10}^{2}\left(\lambda_{j}-\lambda_{j-1}\right)+\lambda_{j}\sigma_{1j}^{2}}:\lambda_{j}\sigma_{1j}^{2}>\left(\lambda_{j-1}-\lambda_{j}\right)\sigma_{10}^{2}\;;\;j\neq 1\right\},\\ A_{8} & =\left\{\frac{\tau^{2}\left(\lambda_{i}Z_{\mathrm{ij}}-\lambda_{i+1}Z_{i+1,j}\right)}{\sigma_{10}^{2}\left(\lambda_{i}-\lambda_{i+1}\right)}:\sigma_{1,i+1}^{2}<\sigma_{1i}^{2}\;;\;i\in \{1,\ldots ,K-1\}/\{j-1,j\}\right\}, \end{array}$$

and we define min({*∅*}) =+ *∞* and max({*∅*}) =− *∞*.

### 4.2 Uniformly minimum variance conditionally unbiased estimator with the closure principle

We can now start to apply our UMVCUE to the example trial setting. In general, assume that we are testing the *K* directional null hypotheses $H_{i}:\mu_{i}\leq \mu_{0}$(*i* = 1,…,*K*) comparing the *K* treatments with the control. Our aim is to strongly control the familywise error rate (FWER) at a pre‐specified level *α*, where strong FWER is defined as the (maximum) probability of rejecting at least one true null hypothesis, irrespective of the configuration of true and false null hypotheses 17.

To control the FWER, we use the *closure principle* (CP) 18. The CP considers all intersection hypotheses that are constructed from the elementary null hypotheses. To strongly control the FWER, an elementary null hypothesis *H*_*i*_ can only be rejected if all intersection hypotheses implying *H*_*i*_ are rejected also. For more details, we refer the reader to the papers of Bretz *et al.* 13, 16. In order to test an intersection hypothesis at the end of a two‐stage trial, we begin by correcting for multiplicity for each stage separately. Only afterwards do we combine the resulting adjusted *p*‐values into a pre‐specified combination function *C*(*p*,*q*). Given an intersection hypothesis *H*_*I*_(where *I*⊆{1,…,*K*}) and the corresponding multiplicity‐adjusted stage 1 *p*‐value *p*_1*I*_ and stage 2 *p*‐value *p*_2*I*_, we reject *H*_*I*_ in the final analysis if $C(p_{1I},p_{2I})\leq c$(where *c* is a suitably chosen critical value to ensure a pre‐specified type I error rate of *α*).

As an example, consider using the closed testing procedure for the stage 1 data with early stopping for futility, using the Bonferonni correction for multiplicity (for the sake of simplicity). The usual first‐stage (unadjusted) *p*‐values for treatment *i*∈{1,…,*K*}, denoted *p*_1,*i*_, are as follows:

$$p_{1,i}=1-\Phi \;\left(\frac{X_{i}-X_{0}}{\sqrt{\sigma_{1i}^{2}+\sigma_{10}^{2}}}\right).$$

For notational convenience, let $r(X_{i})=\frac{X_{i}-X_{0}}{\sqrt{\sigma_{1i}^{2}+\sigma_{10}^{2}}}$ denote the standardised treatment difference for treatment *i*∈{1,…,*K*}.

**Figure 3:**
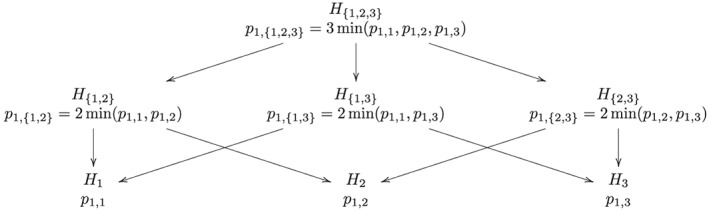
Closed testing procedure for the stage 1 data using the Bonferonni correction, with *K* = 3 treatments.

Consider comparing *K* = 3 treatments with a control (as in our example), as shown in Figure 3.

By the CP, treatment 1 (say) continues to stage 2 if

$$\begin{array}{cc} p_{1,\{1,2,3\}}<\alpha_{0} & \;\Rightarrow \;\underset{i\in \{1,2,3\}}{\max}r(X_{i})>\Phi^{-1}\;\left(1-\alpha_{0}/3\right)\\ p_{1,\{1,2\}}<\alpha_{0} & \;\Rightarrow \;\underset{i\in \{1,2\}}{\max}r(X_{i})>\Phi^{-1}\;\left(1-\alpha_{0}/2\right)\\ p_{1,\{1,3\}}<\alpha_{0} & \;\Rightarrow \;\underset{i\in \{1,3\}}{\max}r(X_{i})>\Phi^{-1}\;\left(1-\alpha_{0}/2\right)\\ p_{1,1}<\alpha_{0} & \;\Rightarrow \;r(X_{1})>\Phi^{-1}\;\left(1-\alpha_{0}\right). \end{array}$$

Without loss of generality, suppose *r*(*X*_1_) > *r*(*X*_2_) > *r*(*X*_3_). Then max*i*∈{1,2,3}*r*(*X*_*i*_) = *r*(*X*_1_), and treatment 1 continues to stage 2 if *r*(*X*_1_) > *Φ* ^−1^(1 − *α*_0_/3). Hence, conditional on the event *Q*={***X***:*r*(*X*_1_) > *r*(*X*_2_) > *r*(*X*_3_),*r*(*X*_1_) > *Φ* ^−1^(1 − *α*_0_/3)}, the UMVCUE for *θ*_1_=*μ*_1_−*μ*_0_ is given by Equation (3), where *K* = 3 and *b* = *Φ* ^−1^(1 − *α*_0_/3).

If treatment 2 also continues to stage 2, then the UMVCUE for *θ*_2_=*μ*_2_−*μ*_0_ is given by Equation (6), where we set *K* = 3, *b*_1_=*Φ* ^−1^(1 − *α*_0_/3), *b*_2_=*Φ* ^−1^(1 − *α*_0_/2) and *b*_3_=−*∞*. Finally, if treatment 3 continues to stage 2, then the UMVCUE for *θ*_3_=*μ*_3_−*μ*_0_ is given by Equation (6), with *K* = 3, *b*_1_=*Φ* ^−1^(1 − *α*_0_/3), *b*_2_=*Φ* ^−1^(1 − *α*_0_/2) and *b*_3_=*Φ* ^−1^(1 − *α*_0_).

### 4.3 Example analysis

Returning to the data from our example trial, we can calculate the stage 1 Bonferroni‐adjusted *p*‐values as given as follows:

$$\begin{array}{cc} p_{1,\{1,2,3\}} & =0.0077\\ p_{1,\{1,2\}}\; & =0.0503,\;p_{1,\{1,3\}}=0.0051,\;p_{1,\{2,3\}}=0.0051\\ p_{1,1}\; & =0.0369,\;p_{1,2}=0.0251,\;p_{1,3}=0.0026. \end{array}$$

Because all of the adjusted *p*‐values are less than *α*_0_, the futility boundary threshold is not crossed for any of the doses, and hence, all of the dose groups (and placebo) are continued to stage 2. Plugging in the observed values (and known variances), the naïve estimator, stage 2 estimator and UMVCUE for the differences between the doses are given in Table 3. We see that the UMVCUE can be higher or lower than both the naïve and stage 2 estimators and is not necessarily closer to the stage 2 data.

**Table 3:** Estimators for the treatment differences from a seamless phase II/III trial.

Stage 1 Rank	Treatment	Naïve	Stage 2	UMVCUE
1	3	2.505	2.200	2.285
2	2	2.250	2.500	2.020
3	1	1.900	2.000	2.062

UMVCUE, uniformly minimum variance conditionally unbiased estimator.

As a brief comparison, the Kimani estimator for the highest ranked treatment is 2.197 using the modified formula shown in Section 3.2. Both these values are lower than the the UMVCUE and the stage 2 estimator, which may be a reflection of the fact that Kimani estimator overcorrects for bias. Note that the Kimani estimator is only for the highest ranked treatment, and estimates for the other treatment differences are unavailable.

Finally, if we want to test the elementary hypotheses *H*_1_,*H*_2_ and *H*_3_ at the end of the trial, we first need to find the stage 2 Bonferroni‐adjusted *p*‐values:

$$\begin{array}{cc} p_{2,\{1,2,3\}} & =0.0216\\ p_{2,\{1,2\}}\; & =0.0144,\;p_{2,\{1,3\}}=0.0307,\;p_{2,\{2,3\}}=0.0144\\ p_{2,1}\; & =0.0233,\;p_{2,2}=0.0072,\;p_{2,3}=0.0153. \end{array}$$

In order to combine the adjusted *p*‐values, we use the well‐known weighted inverse normal combination function *C*(*p*,*q*) = 1−*Φ*[*w*_1_ *Φ* ^−1^(1 − *p*) + *w*_2_ *Φ* ^−1^(1 − *q*)], where the weights *w*_1_,*w*_2_ are set proportional to the originally planned stage‐wise sample sizes: $w_{1}=w_{2}=\sqrt{1/2}$. Because *α*_0_=0.1, then setting *α* = 0.025 means that the critical value *c* = 0.0401.

Taking dose level 3 as an example, by the CP, to reject *H*_3_, we also need to reject the intersection hypotheses *H*_{1,2,3}_,*H*_{1,3}_ and *H*_{2,3}_. Because *C*(*p*_1,{1,2,3}_,*p*_2,{1,2,3}_) < *c*, *C*(*p*_1,{1,3}_,*p*_2,{1,3}_) < *c*, *C*(*p*_1,{2,3}_,*p*_2,{2,3}_) < *c* and *C*(*p*_1,3_,*p*_2,3_) < *c*, then we can indeed reject *H*_3_ and conclude that treatment 3 is superior to placebo. Similarly, following the same procedure, we can also reject *H*_2_ and *H*_1_.

Note that unlike hypothesis testing for the closure principle that strongly controls the type I error regardless of the number of treatments that continue to stage 2, if the estimates obtained after stage 2 are used to select the most effective treatment, all the estimators compared in Table 3 are biased. That is, the estimators derived in this section are only unbiased if the treatments will not be ranked after stage 2. This is because the estimators do not adjust for additional selection at the end of the trial.

## 5 Discussion

In seamless phase II/III trials, it may be desirable to explicitly take into account differences in the precision of the treatment effect estimates. Also, if more than one treatment is taken forward to the second stage, then it is natural to estimate the effect of treatments other than the highest ranked. In this paper, we described a framework for unbiased estimation that is applicable in full generality for the *j*th‐ranked treatment, where the precision of treatment effect estimates can take arbitrary values. Our generalised early stopping rules for futility means that our methodology can be applied where the interim selection rules are driven by formal hypothesis testing procedures, as would be expecting in practice.

Our UMVCUE for the maximum treatment difference is different analytically from the Kimani *et al.* estimator in the special case where the treatment effect variances are equal within each stage. Somewhat counter‐intuitively, our numerical simulations showed that when this special case is satisfied, our UMVCUE is slightly less efficient. The reason is that the Kimani estimator uses all of the data *X*_0_,*X*_1_,…,*X*_*K*_,*Y*_0_,*Y*_1_ explicitly, whereas the UMVCUE uses only the treatment differences *X*_1_−*X*_0_,…,*X*_*K*_−*X*_0_,*Y*_1_−*Y*_0_. Hence, if selection is indeed based on the observed stage 1 sample means, then we would recommend using the Kimani estimator (or its modification when variances in different treatment arms cannot be assumed equal).

When we do in fact have unequal variances and rank by standardised treatment difference, then our simulation results demonstrate how the Kimani estimator overcorrects for bias, because it conditions on different selection rules from those being actually used. This negative bias can be particularly severe when the ratio of the stage 1 treatment variances is greater than 1:2, and in these cases, the MSE of the Kimani estimator increases above the UMVCUE. These results indicate that the difference between the selection rules (and hence the estimators) is greatest when there is reason to believe that the treatment effect variances are different from treatment to treatment, such as when different classes of drugs are being tested and we have variance estimates from pilot studies.

Hence, if selection is based the standardised treatment differences, then we would recommend using the new UMVCUE, because it is unbiased and generally has a lower MSE. Note that our new estimator complements the existing Kimani estimator (and its modification). Indeed, the two frameworks are answering different questions because of the different selection rules being used.

We also showed how to extend our framework to estimate the *j*th‐ranked treatment effect for *j* > 1, in contrast to the Kimani estimator, which is only for the largest treatment difference. Our extended UMVCUE can then be applied within the context of formal hypothesis testing, where we correct for multiplicity with the closure principle. For simplicity, we used the Bonferroni correction in our example, but our framework could also be extended to work with more powerful multiplicity adjustment methods, such as the Simes, Holm or Hochberg procedures (as described in, e.g. 19).

In this paper, we only looked at point estimation of the treatment difference. However, in practice, it is natural to also seek confidence intervals at the end of the study. One possibility would be to use a parametric bootstrap procedure, similar to that described in 20 and 21. Alternatively, it might be possible to adapt the analytic approach of Sampson and Sill 22 to the seamless phase II/III trial setting.

A limitation of our work is that we assume that the variance of the treatment differences are known. In practice, if we have individual variance estimates for each treatment arm, then these will be less precise than a pooled estimate. In order to correctly account for this, one avenue of research is to extend the formulae of Cohen and Sackrowitz 8, who derived the UMVCUE in the independent normal setting (but without the option of early stopping) where the variances are unknown and have to be estimated.

A possible extension is to consider trials where there is early stopping for efficacy. This would be especially compatible with much of the literature on the combination test approach, where there can be early rejection of the null hypothesis 16. However, we anticipate that the UMVCUE would become much more complex in this setting, because of the additional restrictions on the support of *Y*_*j*_. Finally, it is an open question whether there exist UMVCUEs for the treatment differences that are functions of all of the data (***X***,***Y***) instead of just (***X***,*Y*_0_,*Y*_*j*_). If such estimators do exist, then they may outperform both our UMVCUE and the Kimani estimator in terms of MSE.

## Appendix A

### A.1 Derivation of the UMVCUE for the maximum treatment difference

Theorem A.1 The UMVCUE for *θ*_1_=*μ*_1_−*μ*_0_ given *Q* is

(A1) $$Û=\frac{\tau^{2}Z_{1}}{\nu^{2}+\tau^{2}}-\frac{\tau^{2}}{\sqrt{\nu^{2}+\tau^{2}}}\frac{\phi (W_{1})-\phi (W_{2})}{\Phi (W_{1})-\Phi (W_{2})}\;\;,$$

where

$$\begin{array}{cc} W_{i} & =\frac{k_{i}\sqrt{\nu^{2}+\tau^{2}}}{\tau^{2}}-\frac{Z_{1}}{\sqrt{\nu^{2}+\tau^{2}}}\;\text{for}i=1,2\;\;;\\ k_{1} & =\min(A_{1},A_{2},A_{3}),\;k_{2}=\max(A_{4},A_{5}),\\ A_{1} & =\frac{\tau^{2}}{\nu^{2}}\left(Z_{1}-\frac{b}{\lambda_{1}}\right),\\ A_{2} & =\left\{\frac{\tau^{2}\left(\lambda_{1}Z_{1}-\lambda_{2}Z_{2}\right)}{\sigma_{10}^{2}\left(\lambda_{1}-\lambda_{2}\right)+\lambda_{1}\sigma_{11}^{2}}:\lambda_{1}\sigma_{11}^{2}>\left(\lambda_{2}-\lambda_{1}\right)\sigma_{10}^{2}\right\},\\ A_{3} & =\left\{\frac{\tau^{2}\left(\lambda_{j}Z_{j}-\lambda_{j+1}Z_{j+1}\right)}{\sigma_{10}^{2}\left(\lambda_{j}-\lambda_{j+1}\right)}:\sigma_{1,j+1}^{2}>\sigma_{1j}^{2}\;;\;j=2,\ldots ,K-1\right\},\\ A_{4} & =\left\{\frac{\tau^{2}\left(\lambda_{1}Z_{1}-\lambda_{2}Z_{2}\right)}{\sigma_{10}^{2}\left(\lambda_{1}-\lambda_{2}\right)+\lambda_{1}\sigma_{11}^{2}}:\lambda_{1}\sigma_{11}^{2}<\left(\lambda_{2}-\lambda_{1}\right)\sigma_{10}^{2}\right\},\\ A_{5} & =\left\{\frac{\tau^{2}\left(\lambda_{j}Z_{j}-\lambda_{j+1}Z_{j+1}\right)}{\sigma_{10}^{2}\left(\lambda_{j}-\lambda_{j+1}\right)}:\sigma_{1,j+1}^{2}<\sigma_{1j}^{2}\;;\;j=2,\ldots ,K-1\right\}, \end{array}$$

and we define min({*∅*}) =+ *∞*and max({*∅*}) =− *∞*.
Everything follows through as for the multivariate normal setting 15, except that the support of *Y* changes and hence (*k*_1_,*k*_2_) changes too. Conditioning on the event *Q* means that *λ*_*i*_Θ_*i*_>*λ*_*i* + 1_Θ_*i* + 1_ for *i* = 1,…,*K* − 1. Using the equations for the sufficient statistics (2), this gives the following when *i* = 2,…,*K* − 1:

(A2) $$\begin{array}{cc} \lambda_{i}\Theta_{i}>\lambda_{i+1}\Theta_{i+1} & \;\Rightarrow \;\lambda_{i}\left(Z_{i}-\frac{\sigma_{10}^{2}}{\tau^{2}}Y\right)>\lambda_{i+1}\left(Z_{i+1}-\frac{\sigma_{10}^{2}}{\tau^{2}}Y\right)\\ \;\Rightarrow \;\frac{\sigma_{10}^{2}}{\tau^{2}}(\lambda_{i}-\lambda_{i+1})Y<\lambda_{i}Z_{i}-\lambda_{i+1}Z_{i+1}. \end{array}$$

Since $\lambda_{i}=1/\sqrt{\sigma_{1i}^{2}+\sigma_{10}^{2}}$, then $\lambda_{i}>\lambda_{i+1}\;\Leftrightarrow \;\sigma_{1,i+1}^{2}>\sigma_{1i}^{2}$. Hence if $\sigma_{1,i+1}^{2}>\sigma_{1i}^{2}$ then equation (A2) implies that

$$Y<\frac{\tau^{2}(\lambda_{j}Z_{j}-\lambda_{j+1}Z_{j+1})}{\sigma_{10}^{2}(\lambda_{j}-\lambda_{j+1})}.$$

Conversely, if $\sigma_{1,i+1}^{2}<\sigma_{1i}^{2}$ then equation (A2) implies that

$$Y>\frac{\tau^{2}(\lambda_{j}Z_{j}-\lambda_{j+1}Z_{j+1})}{\sigma_{10}^{2}(\lambda_{j}-\lambda_{j+1})}.$$

However, when $\sigma_{1,i+1}^{2}=\sigma_{1i}^{2}$, then there is no restriction contributed to the support of *Y*. Similarly, for *i* = 1 we have

$$\begin{array}{cc} \lambda_{1}\Theta_{1}>\lambda_{2}\Theta_{2} & \;\Rightarrow \;\lambda_{1}\left(Z_{1}-\frac{\sigma_{10}^{2}+\sigma_{11}^{2}}{\tau^{2}}Y\right)>\lambda_{2}\left(Z_{2}-\frac{\sigma_{10}^{2}}{\tau^{2}}Y\right)\\ \;\Rightarrow \;\frac{1}{\tau^{2}}\left[(\lambda_{1}-\lambda_{2})\sigma_{10}^{2}+\lambda_{1}\sigma_{11}^{2}\right]Y<\lambda_{1}Z_{1}-\lambda_{2}Z_{2}. \end{array}$$

Finally, the futility boundary *b* gives the following restriction on the support of *Y*:

$$\begin{array}{cc} \lambda_{1}\Theta_{1}>b & \;\Rightarrow \;\lambda_{1}\left(Z_{1}-\frac{\nu^{2}}{\tau^{2}}Y\right)>b\\ \;\Rightarrow \;Y<\frac{\tau^{2}}{\nu^{2}}\left(Z_{1}-\frac{b}{\lambda_{1}}\right). \end{array}$$

Putting everything together, we have that *k*_1_<*Y* < *k*_2_, where (*k*_1_,*k*_2_) are as in equation (A1). □

### A.2 Derivation of the UMVCUE for the jth ranked treatment difference

Theorem A.2 For a given value of *j*∈{1,…,*K*}, the UMVCUE for *θ*_*j*_=*μ*_*j*_−*μ*_0_ given *Q*′ is

(A3) $${Û}_{j}=\frac{\tau_{j}^{2}Z_{\mathrm{jj}}}{\nu_{j}^{2}+\tau_{j}^{2}}-\frac{\tau_{j}^{2}}{\sqrt{\nu_{j}^{2}+\tau_{j}^{2}}}\frac{\phi (W_{1})-\phi (W_{2})}{\Phi (W_{1})-\Phi (W_{2})}\;\;,$$

where

$$\begin{array}{cc} W_{i} & =\frac{k_{i}\sqrt{\nu_{j}^{2}+\tau_{j}^{2}}}{\tau_{j}^{2}}-\frac{Z_{\mathrm{jj}}}{\sqrt{\nu_{j}^{2}+\tau_{j}^{2}}}\;\text{for}i=1,2\;\;;\\ k_{1} & =\min(A_{1},A_{2},A_{3},A_{4},A_{5}),\;k_{2}=\max(A_{6},A_{7},A_{8}),\\ A_{1} & =\frac{\tau_{j}^{2}}{\nu_{j}^{2}}\left(Z_{\mathrm{jj}}-\frac{b_{j}}{\lambda_{j}}\right),\\ A_{2} & =\left\{\frac{\tau_{i}^{2}}{\sigma_{10}^{2}}\left(Z_{\mathrm{ij}}-\frac{b_{i}}{\lambda_{i}}\right):i\neq j,\;i\in \{1,\ldots ,K\}\right\}\\ A_{3} & =\left\{\frac{\tau_{j}^{2}\left(\lambda_{j}Z_{\mathrm{jj}}-\lambda_{j+1}Z_{j+1,j}\right)}{\sigma_{10}^{2}\left(\lambda_{j}-\lambda_{j+1}\right)+\lambda_{j}\sigma_{1j}^{2}}:\lambda_{j}\sigma_{1j}^{2}>\left(\lambda_{j+1}-\lambda_{j}\right)\sigma_{10}^{2}\;;\;j\neq K\right\},\\ A_{4} & =\left\{\frac{\tau_{j}^{2}\left(\lambda_{j}Z_{\mathrm{jj}}-\lambda_{j-1}Z_{j-1,j}\right)}{\sigma_{10}^{2}\left(\lambda_{j}-\lambda_{j-1}\right)+\lambda_{j}\sigma_{1j}^{2}}:\lambda_{j}\sigma_{1j}^{2}<\left(\lambda_{j-1}-\lambda_{j}\right)\sigma_{10}^{2}\;;\;j\neq 1\right\},\\ A_{5} & =\left\{\frac{\tau^{2}\left(\lambda_{i}Z_{\mathrm{ij}}-\lambda_{i+1}Z_{i+1,j}\right)}{\sigma_{10}^{2}\left(\lambda_{i}-\lambda_{i+1}\right)}:\sigma_{1,i+1}^{2}>\sigma_{1i}^{2}\;;\;i\in \{1,\ldots ,K-1\}/\{j-1,j\}\right\},\\ A_{6} & =\left\{\frac{\tau_{j}^{2}\left(\lambda_{j}Z_{\mathrm{jj}}-\lambda_{j+1}Z_{j+1,j}\right)}{\sigma_{10}^{2}\left(\lambda_{j}-\lambda_{j+1}\right)+\lambda_{j}\sigma_{1j}^{2}}:\lambda_{j}\sigma_{1j}^{2}<\left(\lambda_{j+1}-\lambda_{j}\right)\sigma_{10}^{2}\;;\;j\neq K\right\},\\ A_{7} & =\left\{\frac{\tau_{j}^{2}\left(\lambda_{j}Z_{\mathrm{jj}}-\lambda_{j-1}Z_{j-1,j}\right)}{\sigma_{10}^{2}\left(\lambda_{j}-\lambda_{j-1}\right)+\lambda_{j}\sigma_{1j}^{2}}:\lambda_{j}\sigma_{1j}^{2}>\left(\lambda_{j-1}-\lambda_{j}\right)\sigma_{10}^{2}\;;\;j\neq 1\right\},\\ A_{8} & =\left\{\frac{\tau^{2}\left(\lambda_{i}Z_{\mathrm{ij}}-\lambda_{i+1}Z_{i+1,j}\right)}{\sigma_{10}^{2}\left(\lambda_{i}-\lambda_{i+1}\right)}:\sigma_{1,i+1}^{2}<\sigma_{1i}^{2}\;;\;i\in \{1,\ldots ,K-1\}/\{j-1,j\}\right\}, \end{array}$$

and we define min({*∅*}) =+ *∞* and max({*∅*}) =− *∞*.
The proof is very similar to that for the uniformly minimum variance conditionally unbiased estimator (UMVCUE) for the maximum treatment difference. We just need to determine how the support of *Y*_*j*_ changes. Conditioning on the event *Q*′ means that *λ*_*i*_Θ_*i*_>*λ*_*i* + 1_Θ_*i* + 1_ for *i* = 1,…,*K* − 1. Hence, for *i*∉{*j* − 1,*j*}, we obtain the inequalities corresponding to the sets *A*_5_ and *A*_8_ (in the same way as before). When *i* = *j* and *j* ≠ *K*, we have

$$\begin{array}{cc} \lambda_{j}\Theta_{j}>\lambda_{j+1}\Theta_{j+1} & \;\Rightarrow \;\lambda_{j}\left(Z_{\mathrm{jj}}-\frac{\sigma_{10}^{2}+\sigma_{1j}^{2}}{\tau_{j}^{2}}Y_{j}\right)>\lambda_{j+1}\left(Z_{j+1,j}-\frac{\sigma_{10}^{2}}{\tau_{j}^{2}}Y_{j}\right)\\ \;\Rightarrow \;\frac{1}{\tau_{j}^{2}}\left[\left(\lambda_{j}-\lambda_{j+1}\right)\sigma_{10}^{2}+\lambda_{j}\sigma_{1j}^{2}\right]Y_{j}<\lambda_{j}Z_{\mathrm{jj}}-\lambda_{j+1}Z_{j+1,j}. \end{array}$$

When *i* = *j* − 1 and *j* ≠ 1, we have

$$\begin{array}{cc} \lambda_{j-1}\Theta_{j-1}>\lambda_{j}\Theta_{j} & \;\Rightarrow \;\lambda_{j-1}\left(Z_{j-1,j}-\frac{\sigma_{10}^{2}}{\tau_{j}^{2}}Y_{j}\right)>\lambda_{j}\left(Z_{\mathrm{jj}}-\frac{\sigma_{10}^{2}+\sigma_{1j}^{2}}{\tau_{j}^{2}}Y_{j}\right)\\ \;\Rightarrow \;\frac{1}{\tau_{j}^{2}}\left[\left(\lambda_{j}-\lambda_{j-1}\right)\sigma_{10}^{2}+\lambda_{j}\sigma_{1j}^{2}\right]Y_{j}>\lambda_{j}Z_{\mathrm{jj}}-\lambda_{j-1}Z_{j-1,j}. \end{array}$$

Finally, the early stopping rules for futility means that *λ*_*i*_Θ_*i*_>*b*_*i*_ for *i* = 1,…,*K*. When *i* ≠ *j*, this means that $\lambda_{i}\left(Z_{\mathrm{ij}}-\frac{\sigma_{10}^{2}}{\tau_{j}^{2}}Y_{j}\right)>b_{i}\;\Rightarrow \;Y_{j}<\frac{\tau_{j}^{2}}{\sigma_{10}^{2}}\left(Z_{\mathrm{ij}}-\frac{b_{i}}{\lambda_{i}}\right)$, whilst when *i* = *j*, this implies that $\lambda_{j}\left(Z_{\mathrm{jj}}-\frac{\nu_{j}^{2}}{\tau_{j}^{2}}Y_{j}\right)>b_{j}\;\Rightarrow \;Y_{j}<\frac{\tau_{j}^{2}}{\nu_{j}^{2}}\left(Z_{\mathrm{jj}}-\frac{b_{j}}{\lambda_{j}}\right)$. Putting everything together, we have that *k*_1_<*Y* < *k*_2_, where (*k*_1_,*k*_2_) are as in Equation (A3). □

## References

[1] Barnes PJ , Pocock SJ , Magnussen H , Iqbal A , Kramer B , Higgins M , Lawrence D . Integrating indacaterol dose selection in a clinical study in COPD using an adaptive seamless design. Pulmonary Pharmacology & Therapeutics 2010; 23(3): 165–171. 20080201 10.1016/j.pupt.2010.01.003

[2] Zeymer U , Suryapranata H , Monassier JP , Opolski G , Davies J , Rasmanis G , Linssen G , Tebbe U , Schroeder R , Tiemann R , Machnig T , Neuhaus KL . The Na+/H+ exchange inhibitor eniporide as an adjunct to early reperfusion therapy for acute myocardial infarction. Results of the evaluation of the safety and cardioprotective effects of eniporide in acute myocardial infarction (ESCAMI) trial. Journal of the American College of Cardiology 2001; 38(6): E1644–E1650. 10.1016/s0735-1097(01)01608-4 11704395

[3] Schmoll H , Cunnighmam DAS , Krapetis C , Rougier P , Koski SPB , Mookerjee B , Robertson J , van Cutsem E . mFOLFOX6 + cediranib vs mFOLFOX6 + bevacizumab in previously untreated metastatic colorectal cancer (MCRC): a randomised, doubleblind, phase II/III study (HORIZON III). Annals of Oncology 2010; 21(suppl 8): viii189–viii224.

[4] EMA . CHMP reflection paper on methodological issues in confirmatory clinical trials planned with an adaptive design 29 March 2016. http://www.ema.europa.eu/docs/en_GB/document_library/Scientific_guideline/2009/09/WC500003616.pdf.

[5] FDA . Draft guidance for industry adaptive design clinical tials for drugs and biologics 29 March 2016. http: //www.fda.gov/downloads/Drugs/.../Guidances/ucm201790.pdf.

[6] Bauer P , Koenig F , Brannath W , Posch M . Selection and bias – two hostile brothers. Statistics in Medicine 2010; 29(1): 1–13. 19844944 10.1002/sim.3716

[7] Kimani PK , Todd S , Stallard N . Conditionally unbiased estimation in phase II/III clinical trials with early stopping for futility. Statistics in Medicine 2013; 32(17): 2893–2910. 23413228 10.1002/sim.5757 PMC3813981

[8] Cohen A , Sackrowitz HB . Two stage conditionally unbiased estimators of the selected mean. Statistics & Probability Letters 1989; 8(3): 273–278.

[9] Bowden J , Brannath W , Glimm E . Empirical Bayes estimation of the selected treatment mean for two‐stage drop‐the‐loser trials: a meta‐analytic approach. Statistics in Medicine 2014; 33(3): 388–400. 23873666 10.1002/sim.5920 PMC4282323

[10] Bowden J , Glimm E . Unbiased estimation of selected treatment means in two‐stage trials. Biometrical Journal 2008; 50(4): 515–527. 18663760 10.1002/bimj.200810442

[11] Bowden J , Glimm E . Conditionally unbiased and near unbiased estimation of the selected treatment mean for multistage drop‐the‐losers trials. Biometrical Journal 2014; 56(2): 332–349. 24353149 10.1002/bimj.201200245 PMC4034592

[12] Carreras M , Brannath W . Shrinkage estimation in two‐stage adaptive designs with midtrial treatment selection. Statistics in Medicine 2013; 32(10): 1677–1690. 22744936 10.1002/sim.5463

[13] Bretz F , Schmidli H , König F , Racine A , Maurer W . Confirmatory seamless phase II/III clinical trials with hypotheses selection at interim: general concepts. Biometrical Journal 2006; 48(4): 623–634. 16972714 10.1002/bimj.200510232

[14] Stallard N , Todd S . Seamless phase II/III designs. Statistical Methods in Medical Research 2011; 20(6): 623–634. 20724313 10.1177/0962280210379035

[15] Robertson DS , Prevost AT , Bowden J . Accounting for selection and correlation in the analysis of two‐stage genome‐wide association studies. Biostatistics. Advance online publication. DOI: 10.1093/biostatistics/kxw012. 10.1093/biostatistics/kxw012 PMC5031943 26993061

[16] Bretz F , Koenig F , Brannath W , Glimm E , Posch M . Adaptive designs for confirmatory clinical trials. Statistics in Medicine 2009; 28: 1181–1217. 19206095 10.1002/sim.3538

[17] Hochberg Y , Tamhane AC . Multiple Comparison Procedures. Wiley: New York, 1987.

[18] Marcus R , Eric P , Gabriel KR . On closed testing procedures with special reference to ordered analysis of variance. Biometrika 1976; 63(3): 655–660.

[19] Alosh M , Bretz F , Huque M . Advanced multiplicity adjustment methods in clinical trials. Statistics in Medicine 2013; 33: 693–713. 24105821 10.1002/sim.5974

[20] Bowden J , Dudbridge F . Unbiased estimation of odds ratios: combining genomewide association scans with replication studies. Genetic Epidemiology 2009; 33(5): 406–418. 19140132 10.1002/gepi.20394 PMC2726957

[21] Pepe MS , Feng Z , Longton G , Koopmeiners J . Conditional estimation of sensitivity and specificity from a phase 2 biomarker study allowing early termination for futility. Statistics in Medicine 2009; 28: 762–779. 19097251 10.1002/sim.3506 PMC2745932

[22] Sampson AR , Sill MW . Drop‐the‐losers design: normal case. Biometrical Journal 2005; 47(3): 257–268. 16053251 10.1002/bimj.200410119

